# Brachiaria species influence nitrate transport in soil by modifying soil structure with their root system

**DOI:** 10.1038/s41598-020-61986-0

**Published:** 2020-03-19

**Authors:** M. V. Galdos, E. Brown, C. A Rosolem, L. F. Pires, P. D. Hallett, S. J. Mooney

**Affiliations:** 1 Institute for Climate and Atmospheric Science, School of Earth and Environment, University of Leeds, Leeds, LS2 9JT UK; 2 Division of Agricultural & Environmental Science, School of Biosciences, University of Nottingham, Sutton Bonington Campus, Sutton Bonington, Loughborough, LE12 5RD UK; 3 Department of Crop Science, São Paulo State University, Botucatu, Brazil; 4 Department of Physics, State University of Ponta Grossa, Ponta Grossa, Brazil; 5 School of Biological Sciences, University of Aberdeen, Aberdeen, UK

**Keywords:** Plant sciences, Environmental sciences

## Abstract

Leaching of nitrate from fertilisers diminishes nitrogen use efficiency (the portion of nitrogen used by a plant) and is a major source of agricultural pollution. To improve nitrogen capture, grasses such as brachiaria are increasingly used, especially in South America and Africa, as a cover crop, either via intercropping or in rotation. However, the complex interactions between soil structure, nitrogen and the root systems of maize and different species of forage grasses remain poorly understood. This study explored how soil structure modification by the roots of maize (*Zea* maize), palisade grass (*Brachiaria brizantha* cv. Marandu) and ruzigrass (*Brachiaria ruziziensis*) affected nitrate leaching and retention, measured via chemical breakthrough curves. All plants were found to increase the rate of nitrate transport suggesting root systems increase the tendency for preferential flow. The greater density of fine roots produced by palisade grass, subtly decreased nitrate leaching potential through increased complexity of the soil pore network assessed with X-ray Computed Tomography. A dominance of larger roots in ruzigrass and maize increased nitrate loss through enhanced solute flow bypassing the soil matrix. These results suggest palisade grass could be a more efficient nitrate catch crop than ruzigrass (the most extensively used currently in countries such as Brazil) due to retardation in solute flow associated with the fine root system and the complex pore network.

## Introduction

Globally, high rates of nitrogen fertiliser are applied as a means to increase crop yields. Incomplete plant uptake and high rainfall can cause soluble nitrate ions to be leached out of the rooting zone and into the groundwater, diminishing nitrogen use efficiency i.e. the portion of applied nitrogen that is taken up and used by a plant^1^. The annual financial cost of nitrate fertiliser loss from agricultural land has been estimated at $210B in the US^2^ and €230B in Europe^3^, with knock-on environmental costs from groundwater contamination. Similar estimates are unavailable for Brazil but Pires *et al*.^4^ suggested a modest 2% increase in NUE could equate to saving of $21 M in N fertilizer costs. Nitrate leaching is intensified by agriculture, with drainage channels and shallow aquifers underneath intensively managed agricultural land most prone to nitrate pollution^5^. An excess of nitrate in groundwater can lead to significant ecological implications such as eutrophication and impacts to human health^6^.

Controlling nitrate leaching from agricultural fields has been an active area of research for decades^7–9^. The European Water Framework Directive identifies Nitrate Vulnerable Zones where nitrate leaching is prevalent and seeks to provide management interventions at a local scale^10^. However, there are significant legislation gaps on issues such as pollution and water stress in Brazil^11^. Improved precision in fertiliser application, *in situ* sensing and optimising crop selection offer great potential in reducing the risk of nitrate leaching. Previous studies have focused on nitrate removal methods including biochar application to increase the cation exchange capacity^12^.

It is well known that the root architecture of crop plants can alter soil structure^13,14^, strongly influencing important characteristics such as the hydraulic properties of soil^15^ and nitrogen transformations^16^. However, few studies have directly considered the effect of a plant’s root system architecture on the leaching potential of nutrients in soil. Macleod *et al*.^17^ showed that hybrid grass species can be used to enhance water infiltration rates by as much as 50% compared with common varieties, which they attributed to a rapid initial root growth. Dunbabin *et al*.^18^ proposed that high root density in the topsoil may increase both nitrate and water uptake by plants, reducing nitrate leaching. Conversely, other studies suggest that due to the high mobility of the nitrate ion, nitrate acquisition does not require high rooting densities^19^. Scope exists to adopt agronomic practices that enhance nitrate capture and decrease losses, either by selecting crop root systems capable of capturing deep^20^ or diffuse^18^ nitrate pools, or that can alter hydrological properties through changes to soil physical structure^21^. This is reflected in large-scale land management changes of the fragile soils of the Brazilian Cerrado, an agricultural area particularly at risk from nitrate leaching due to intensive agricultural practises^22^.

Management techniques used to prevent soil and nutrient losses in the Cerrado include intercropping with forage species, zero tillage and contour banks^23^. The use of brachiaria grass species in crop-forage intercropping systems has been recently adopted across Brazil. Intercropping and crop rotations have been shown to reduce losses from applied mineral nitrate and support larger maize yields due to negligible competition between the maize and brachiaria^24^. These systems also provide increased returns from the land through cattle forage^25^, providing both environmental and economic benefits. However, it is not known at present whether the brachiaria species that are currently selected are the most effective at minimising nitrate losses, either through uptake or improved storage in the soil. In this case, the nitrogen use efficiency will be driven by interactions between the root systems of maize and brachiaria within the soil environment. Through the use of non-invasive imaging and traditional leaching experiments, it is possible to disentangle the interactions between root systems and their impacts on soil structure^26^, and the transport and retention of nitrate in soil^27^. This investigation assessed the effect of the root systems of maize and two brachiaria species, ruzigrass (*Brachiaria ruziziensis*), the most commonly used species in Brazil, and palisade grass (*Brachiaria brizantha* cv. Marandu) on nitrate leaching using a controlled environment experiment with repacked soil cores. We hypothesised that i) plant root systems have a significant impact on nitrate leaching via manipulation of the soil porous architecture and ii) that species with roots systems that enhance the complexity of the pore network through an increased generation of the smallest sized and well connected pores would decrease nitrate leaching by increase nitrate residence time. The research aimed to help guide crop selection and intercropping practices to improve nitrogen use efficiency, in terms of the uptake ability of the plant via the soil system, and thus seeking to decrease losses of nitrogen to the environment.

## Materials and Methods

### Soil Column Preparation

Samples of Typic Rhodudalf (distroferric Red Nitosol) soil was collected from the Sao Paulo State University experimental farm in Botucatu, Sao Paulo State, Southeastern Brazil (22.49°S, 48.25°W). Soil samples were collected to a depth of 0.3 m from a long-term crop-rotation experiment^28^ and shipped to the UK for the leaching and imaging experiments. The soil has a clay texture and selected physical and chemical characteristics are presented in Table 1.

**Table 1:** Selected soil physical and chemical characteristics.

Parameter	Unit	Value
Clay (<0.002 mm)	g kg^−1^	614
Silt (0.05–0.002 mm)	g kg^−1^	239
Sand (2.00–0.05 mm)	g kg^−1^	147
pH (CaCl_2_)		4.9
Organic Carbon	g kg^−1^	21.7
P	mg dm^−3^	70.6
Ca	mmolc dm^−3^	54.3
Mg	mmolc dm^−3^	15.7
K	mmolc dm^−3^	4.2
Al + H	mmolc dm^−3^	55.5
CEC	mmolc dm^−3^	68.1
Base Saturation	V%	57.2
Total Nitrogen	%	0.15

Soil columns were prepared using 6.5 cm diameter × 12 cm length polyvinyl chloride pipes, with the bottom end tightly covered with a muslin cloth. The soil samples were air dried, sieved to 2 mm, and packed into the columns at a bulk density of 1.2 g cm^−3^, leaving 1 cm headspace at the top of the column for leaching experiments. This bulk density is representative of typical topsoil field conditions for this soil. The columns were saturated for 24 hours before being drained for 48 hours to reach a notional field capacity before seedlings were planted.

Maize (Cv. F1 Golden Mountain) (*Zea mays*), ruzigrass (*Brachiaria ruziziensis* R. Germ. and C.M. Evrard), and palisade grass [*Brachiaria brizantha*, (Hochst. Ex A. Rich.) Stapf. – cultivar Marandu] were used in this study. The seeds were germinated 5 days prior to planting. The maize columns were planted at a seedling rate of 1 seedling per core, and brachiaria columns at 4 seedlings per core, to match typical field sowing rates, with four replicates per treatment. Sulfuric acid scarification treatment was required to break the dormancy of the brachiaria seeds, involving a 10 and 5-minute sulphuric acid bath for palisade grass and ruzigrass respectively. Once planted the soil columns (along with unplanted controls) were kept in a controlled environment room with a 25 °C day-time temperature, 20 °C night-time temperature and a 12 hour photoperiod for 12 weeks. The notional ‘field capacity’ water content of the cores was maintained for the duration of the experiment by weighing and watering to the initial weight at two-day intervals.

### Breakthrough curves

Nitrate breakthrough curves were obtained using the nitrate pulse method described by Bawatharani *et al*.^29^. The soil columns were saturated for 24 hours prior to the nitrate leaching experiments to ensure all pores were water conducting. A peristaltic pump was used to deliver water to the column. The columns were initially flushed with two times the total pore volume with distilled water to remove residing nitrate ions and to obtain a constant flux. A nitrate pulse was then applied as 10 ml of 0.36 M KNO_3_ (equivalent to a 150 kg N ha^−1^ rate), followed by a continuous flux of distilled water, applied at a rate of 2.0 ml min^−1^. Each core had a pore volume of 200 ml, so it took approximately 100 min for the equivalent of a complete pore volume to flow through the soil. Leachate samples (10 ml) were collected from the bottom of the soil column using a fractional collector at 5 minute intervals for approximately 7 hours, totalling 85 samples per column. The leachate samples were analysed for nitrate concentration using a nitrate ion-sensitive probe (Mettler Toledo), measuring electronic conductivity (mV). This was calibrated using prepared standards of 0.1, 1, 10, 100 and 1000 ppm KNO_3_, repeated before each leaching event. A log-normal regression was used to create a conversion model between the calibration curve and the probe reading. Nitrate breakthrough curves were then plotted against pore volume to assess nitrate transport over time.

### X-ray Computed Tomography (CT)

The columns were drained for 24 h before undergoing non-destructive 3D X-ray imaging using a G.E. v|Tomex|m Micro Computed Tomography (CT) X-ray scanner at the Hounsfield Facility (The University of Nottingham, Sutton Bonington Campus, Leicestershire, UK). The drainage prior to scanning was undertaken as the attenuation of the X-ray beam for roots and water filled pores are similar^30^ and drainage for 24 h usually enhances the root segmentation process^31^. Each soil core was scanned for 1 h 40 m at 160 kV at a resolution of 45 μm, with 2900 collected images per scan. Whilst considerably faster scanning is possible, this longer scan setting was chosen to maximise image quality to aid image processing procedures.

For root segmentation, a combination of RooTrak^32^ for automatic detection, especially of the larger roots, supplemented by the region grower tool in VGStudio MAX 2.2.2, mainly for the finer roots, was used. Then, the 3-D root visualisations were rendered and measurements of root volume and associated characteristics were performed using RooTrak^32^. Once the root material was segmented, the soil pore space was also subjected to a similar assessment. To avoid edge effects that may arise from X-ray beam hardening, soil packing and root growth, a region of interest of 36 mm × 36 mm × 20 mm was cropped from the centre of the core which was maintained for all columns. The segmented root material was converted into a mask and deleted from the image data set so that only soil material was remaining. The images were then processed using ImageJ 1.42 software^33^ (http://rsbweb.nih.gov/ij/). Visual analysis was carried out on each sample before segmentation. The segmentation process was based on the non-parametric Otsu method of automatic thresholding^34^. This process resulted in a binary image in which pores and soil solid material were respectively represented by white (0) and black (255) pixels. Once binarised, the images were used to calculate the total porosity in each image (referred to as ‘image derived porosity’) and the relative size, shape and connection of each pore in 3D using the BoneJ plugin within ImageJ.

The 3D pore size distribution (sorted by pore volume) corresponds to the total number of disconnected volumes of pore space inside the total sample volume, not including the root volume. Pores were classified in three different volume intervals: 0.0007–0.001; 0.001–0.1; >0.1 mm^3^ selected based on the importance of different pore sizes for water movement and retention, i.e. smaller pores function predominantly for water storage whereas larger pores contribute to flow processes. The soil pores were also classified according to their shape using the terminology suggested by Bullock *et al*.^35^, described in detail in Galdos *et al*.^36^. The network tortuosity (τ) of the pores and connectivity was calculated using Osteoimage^37,38^. Tortuosity was determined through the geodesic reconstruction algorithm implemented by Roque *et al*.^39,40^. Geometrically, tortuosity is defined as (Eq. 1):

1 $${\rm{\tau }}=\frac{{\rm{LG}}}{{\rm{LE}}}$$

where LG and LE represent the geodesic length between two connected points within the pore space and the Euclidian length between these two points.

The Euler-Poincaré characteristic (EPC) was utilized to estimate the degree of connectivity of the soil pore system. This parameter for a 3D structure is related to the number of isolated parts minus the connectivity of an object^41^. The EPC number is an indicator of how connected a pore is: the smaller (more negative) it is, the greater the pore connectivity^38,42^.

### Root washing

Following X-ray imaging, the roots were extracted by washing to remove the soil as outlined by Watt *et al*.^43^ and scanned using a flatbed scanner with WinRHIZO software to produce a 2D image. The WinRHIZO derived images were used to estimate total root volume, root surface area, root length, average root diameter and the number of roots with diameters <0.5 mm. Fresh and dried root biomass was also measured for each treatment. Fresh root and shoot biomass samples were weighed, dried for 24 hours at 80 °C, and then re-weighed.

### Soil total nitrogen

The total nitrogen concentration was measured on a composite sample of soil from each column after they had been dismantled for root analysis. Total Nitrogen was measured using a Carbon Nitrogen Analyser (CE instruments, Wigan, UK, Flash EA1112 series). The soil samples were oven dried, finely milled using a ball mill and 20 mg of each sample was used for analysis.

### Statistical analysis

Statistical analysis was performed using the R software^44^ (R version 3.4.3.). The timing and peak of the breakthrough was calculated. The maximum nitrate concentration was taken as the height of the peak of the average breakthrough curve. Its standard deviation was calculated using the nitrate values of the replicate data sets at the same pore volume as the peak in the average curve. The trapezium rule (Eq. 2) was used to approximate the area,

2 $${\int }_{{x}_{0}}^{{x}_{n}}f(x)dx=1/2h[({y}_{0}+{y}_{n})+2({y}_{1}+\cdots +{y}_{n-1})],$$

where x_0_ and x_n_ are the initial and final pore volumes (cm^3^), respectively; y_0_ and y_n_ are the nitrate concentrations (ppm) of the initial and final pore volumes respectively; and h is the x-axis interval (10 ml). Prior to statistical analysis all data was tested to verify normality and homoscedasticity, respectively. Following this an analysis of variance (ANOVA) was performed to test the effects of different treatments on nitrate leaching, plant and soil parameters. Where the ANOVA results were significant (p < 0.05), the means were compared according to Tukey’s post-hoc test (p < 0.05) using the R software. Standard errors of the means were calculated and provided as required.

## Results

### Nitrate breakthrough curves

**Figure 1:**
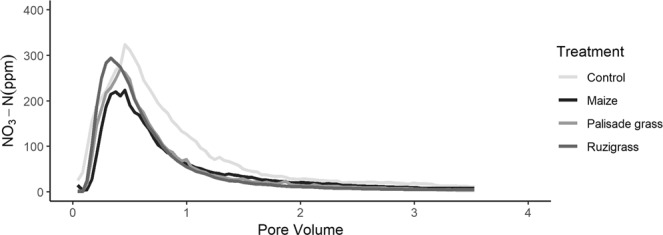
Average brakethrough curve for each treatment as NO_3_-N concentration (ppm) per pore volume leached. N = 4 per treatment. Mean SD in NO_3_-N ppm = Control (39.9); Palisade grass (21.5); Ruzigrass (15.4); Maize (9.96).

The 2.0 ml min^−1^ flow rate imposed by the peristaltic pump was slow enough to prevent ponding when measuring leaching characteristics. Efflux rate from the cores therefore remained constant throughout the experiments. The nitrate breakthrough curves are presented as nitrate concentration per pore volume (Fig. 1). The asymmetric shape and rapidly peaking breakthrough curves indicate that preferential flow i.e. where a portion of the soil pore space is bypassed, was the dominant flow mechanism. This might be expected where a well-connected macropore network has formed that enables solute transport to bypass significant amounts of the soil matrix where either smaller and/or unconnected pores occur. As the soil cores were maintained at a notional field capacity during plant growth, followed by saturation before the leaching experiment, the presence of significant macropore cracks in the soil that might have developed during drying can be discounted, although some small cracks were observed in the X-ray images, these were at the sites of lateral root development only.

**Figure 2:**
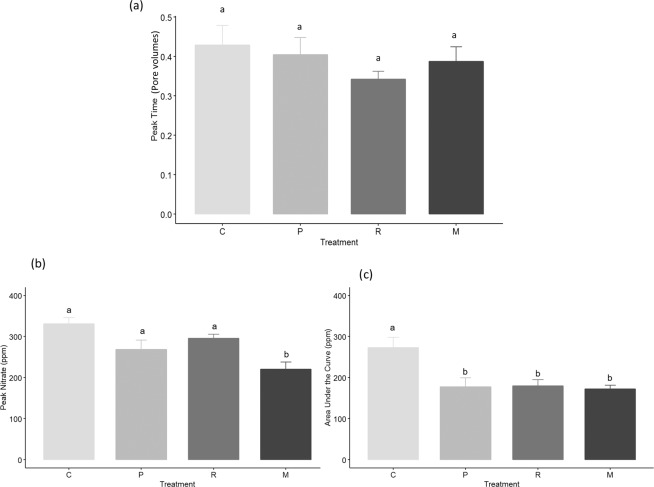
Characteristics of the breakthrough curves; (**a**) peak breakthrough time, (**b**) peak nitrate and (**c**) area under the curve (**b**). Error bars are one standard error from the mean and grouping data is from Tukey pairwise comparisons (95% confidence). C = Control; P = Palisade grass; R = Ruzigrass; M = Maize.

When assessed visually, the control treatment had a retarded breakthrough compared to the planted treatments confirming the first hypothesis. There were small but very clear differences between the shape of the breakthrough curves of the two brachiaria species, and a greater difference between the brachiarias species and maize, which had the lowest nitrate peak. The control treatment reached peak nitrate concentration at 0.43 pore volumes, which was the greatest, whereas ruzigrass, the lowest, reached it within 0.34 pore volumes, equivalent to a time difference of 10 min (Fig. 2a). The treatment differences for time to peak nitrate were significant at *p* = 0.1 but not at *p* = 0.05 (F = 3.327, *p* = 0.0648) due to the within treatment variability. The specific height of the breakthrough, was significantly smaller for the maize treatment (F = 6.23, *p* = 0.0117, Fig. 2b) compared to the other treatments including the control. Whereas the total nitrate leached (area under curve, Fig. 2c) showed significant differences between the control and the plant treatments (F = 6.303, *p* = 0.0113), but there were no significant difference between the maize and brachiaria treatments or between brachiaria treatments. Overall, the control produced a higher but delayed nitrate peak, suggesting the inclusion of plants increased the speed of nitrate leaching but reduced overall nitrate losses. Maize produced the lowest nitrate peak, whilst palisade grass best delayed the nitrate peak potentially increasing the residence time.

### Soil nitrogen

No significant difference in nitrogen concentration was found between treatments (Supplementary Figure 1) as expected as the measurements were taken following flushing the soil columns with the equivalent of four pore volumes of water.

### Root system characteristics

**Figure 3:**
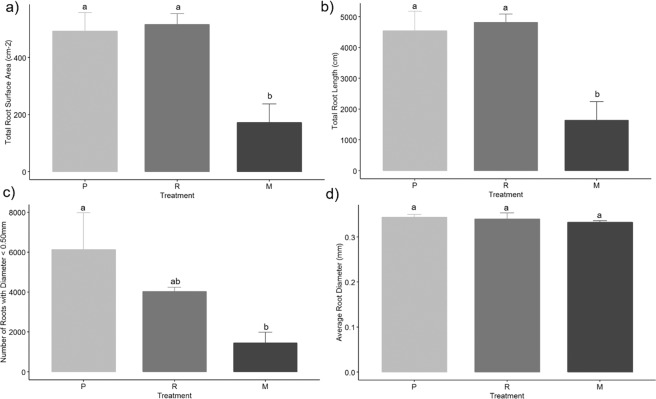
Total root surface area (**a**), root length (**b**), number of roots with diameter <0.5 mm (**c**) and average root diameter (**d**). Error bars are one standard error from the mean. Grouping data from Tukey pairwise comparisons (95% confidence). P = Palisade grass; R = Ruzigrass; M = Maize.

**Figure 4:**
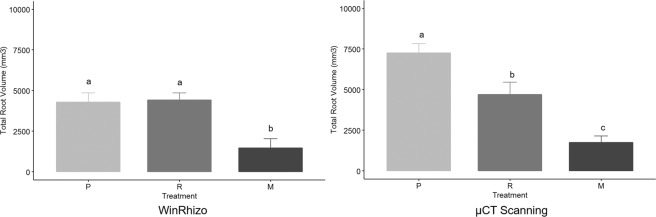
Total root volume in mm^3^ for *B. brizantha*, *B. ruziziensis* and Maize, as calculated from WinRHIZO (left) and µCT scans (right). P = Palisade grass; R = Ruzigrass; M = Maize.

**Figure 5:**
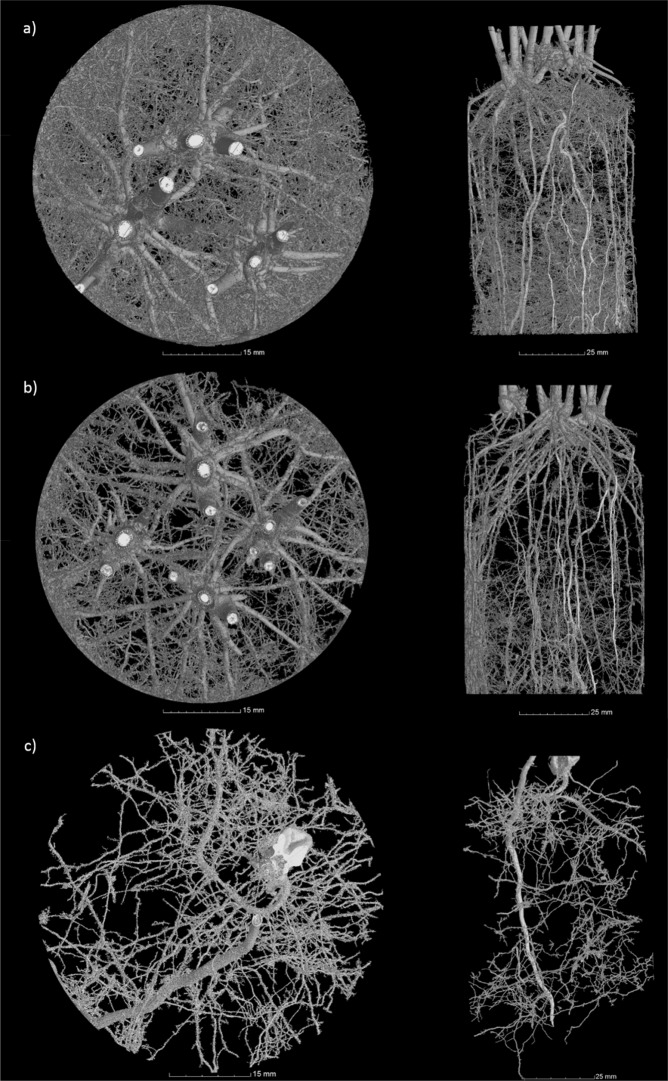
Example xy and zx view images from X-ray Computed Tomography scans of Palisade grass (**a**), Ruzigrass (**b**) and Maize (**c**).

**Figure 6:**
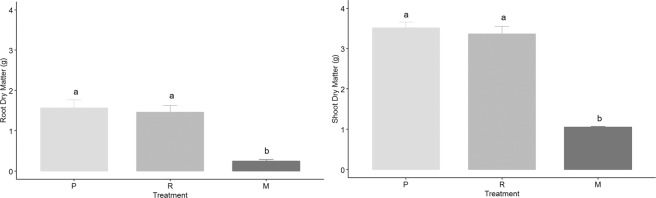
Dried weight of root (left) and shoot (right) biomass for all vegetation treatments. Error bars are one standard error from the mean. Grouping data from Tukey pairwise comparisons (95% confidence). P = Palisade grass; R = Ruzigrass; M = Maize.

Both brachiaria species had a greater total root length, surface area, diameter, volume and dry weight when compared with maize (Figs. 3–6, Supplementary Figure 2). The brachiaria grasses also had a greater number of fine roots (diameter <0.5 mm) in comparison to maize. The root measurements from WinRHIZO (Fig. 3) showed clear significant differences for the number of roots with a diameter <0.50 mm between each brachiaria species and maize and for root length, surface area and total volume between the brachiarias and maize. However, no significant difference was found between plants for root diameter. There were clear differences between the root volumes calculated from the CT and WinRHIZO scans (Fig. 4). The root volume estimated by WinRHIZO was generally less than that determined by CT, especially for the palisade grass due to its fine root system which was approximately one third greater by CT; most likely due to root loss when they were washed from soil. Root volume was significantly different between the three plants via CT imagery following the trend palisade grass > ruzigrass > maize. CT images (Fig. 5) support this finding clearly showing the very extensive root system architectures in brachiaria grasses and a clear difference in fine root fractions between ruzigrass and palisade grass.

### Soil pore network characteristics

**Figure 7:**
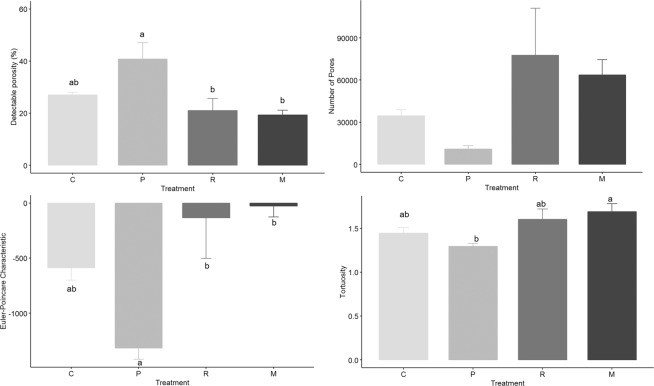
Detectable porosity (%), number of pores, connectivity (Euler-Poincare Characteristic) and tortuosity. Error bars are one standard error from the mean. Grouping data from Tukey pairwise comparisons (95% confidence). C = Control; P = Palisade grass; R = Ruzigrass; M = Maize.

The detectable soil porosity was enhanced significantly by palisade grass over other species and the control (Fig. 7). In addition to increasing the overall porosity, there was a clear decrease in the number of pores created by the palisade grass treatment, this was especially apparent for the largest pore size class of >1 mm^3^ (Supplementary Figure 3). Palisade grass was also responsible for greater complexity in the pore network, as evidenced by the lower (i.e. more negative) EPC value. This equates to greater pore connectivity, and lower tortuosity, which indicates more connected and aligned pores supporting our second hypothesis. Significant differences were also observed in the soil pore shape between the treatments, and especially between palisade grass and maize (Supplementary Figures 4 and 5). Palisade grass promoted the development of more complex and cavernous shaped pores compared to thinner, more crack-shaped pores that were observed with ruzigrass.

## Discussion

Nitrate breakthrough curves varied between the plant treatments (Figs. 1 & 2), which can be explained by the root system architectures shown in Fig. 5. Peak nitrate was significantly different between the highest, recorded in the control, compared to the maize treatment (the lowest). The lower peak for nitrate in maize was likely due to a less prolific root structure in comparison to the brachiaria planted soil columns (Fig. 5). Maize has a rooting structure dominated by thicker main roots^45^ and less lateral roots than brachiaria, although it is important to acknowledge the early growth stage the plants were assessed at in this study. Whilst the differences in the root system architecture between the planted treatments are clear, they are less pronounced in the breakthrough curves, especially between the brachiaria treatments which can be most likely attributable to the high variability that is often observed during soil hydraulic measurements^46^. In addition, our experimentation was performed on a heavy clay soil from Brazil that presents further challenges for hydraulic analysis in terms of establishing constant flow rates and avoiding ponding.

These results highlight the importance of root channels in controlling solute transport through the soil, shown in Fig. 2, and the important role of different rooting structures in this regard, which has not been considered previously. Increased rooting can enhance pore connectivity as we have shown here, which can impact on preferential flow^47^. Roots increase porosity via the creation and expansion  of biopores^48,49^, which are especially effective in clay soil due to enhanced aggregation^50^. Cylindrical macropores can remain in the soil long after the plant has died, depending on the soil texture, which can provide rapid transport pathways for solutes and are resistant to weathering and compaction stresses^51^.

At the root-soil interface, changes in pore structure and potentially the deposition of mucilage and exudates, can enhance hydraulic conductivity around a live root^52^. Plant water uptake around the root may enhance the frequency and extent of repeated wetting and drying of the soil, which could further impact on the macropore distribution and connectivity^53^. In soils with brachiaria, due to their very extensive root system, this is likely to be important. Significant cracking was not observed in this study due to the maintenance of the soil moisture status. However, where it proliferates, the soil is likely to produce preferential flow pathways, especially in clay soils which increases the susceptibility to leaching of nitrate^54^.

Although biopore development will ultimately lead to greater macroporosity, using the pore size distribution on its own to describe solute transport is fraught with uncertainty. It is well known that decreasing the average pore size in soil will decrease hydraulic conductivity and drainage under more negative water potentials^55^, but the shape and connectivity of the pore space is also important. In this study, we hypothesised that the enhanced fine roots in the palisade grass treatment contributes to a delay in the nitrate peak in comparison with the ruzigrass grass treatment (seen in Figs. 1 and 2a), though not significantly different at *p* = 0.05. We observed that palisade grass was associated with enhanced porosity (but a reduced number of pores in the largest size category; Supplementary Figure 3) and an increase in the complexity and irregularity of the pore network. This is likely to impact on solute transport more than the pore size^56^ and a possible reason for the retarded nitrate breakthrough of palisade grass compared to ruzigrass.

The effects of nitrate leaching are typically most pronounced in intensive agricultural areas, although the mechanism by which nitrate moves through the soil is unclear. Our results lead to a new hypothesis that palisade grass is a more suitable cover crop in the Brazilian context examined here, reducing and delaying the nitrate peak in comparison with ruzigrass through generation of a more complex soil pore network which retards the transport of nitrate, increasing its residence time. We suggest this is due to a combination of a more extensive root system, especially at the finer scale, and an enhanced soil pore network both in size and complexity as a result of the root development. Management strategies to enhance N recovery from soil are increasingly sought after. Intercropping, in particular, is a very popular soil management technique in Brazil, especially in the Cerrado region, and usually intercropping with a singular vegetation type is performed, e.g. grasses. However, Scherer-Lorenzen *et al*.^57^ found that total nitrogen loss was also dependent on the species richness and composition. Perego *et al*.^58^ and Zavattaro *et al*.^59^ both reported significant reductions in nitrate leaching in maize and rye grass cropping systems, while the use of leguminous cover crops increased nitrate loss through the continuous supply of available nitrate through biological N fixation^60^.

Our study explored saturated conditions in the absence of plant transpiration so that the interactions between root architecture, the impact of roots on soil structure and nitrate leaching could be disentangled. There is considerable scope for further research under field conditions, including in unsaturated soil^21^. In particular, the influence of different root systems in intercropped systems on nitrogen cycling, capturing nitrogen, as well as affecting nitrate transport through pore structure changes, needs to be explored. Fine roots enable increased water uptake^61^ and are important regulators of biogeochemical cycles including carbon and nitrogen. Increased soil respiration from fine root production was observed by Hendricks *et al*.^62^ suggesting the soil microbial communities benefit from increased fine root turnover.

This study was limited to small cores to enable X-ray CT scanning at a micron scale resolution so that macropores and plant roots from the same sample (that had also been subject to a tracer experiment) could be readily resolved. The resulting root structures created experimental artefacts, especially in the brachiaria species, as roots became pot bound quite quickly. It is possible that this root architecture may have contributed to the increased potential for preferential flow. In addition, due to the limited length of the column, the effects of final rooting depth on nitrate losses could not be assessed. Moreover, as biopores terminate in the soil at depth, even though they may enhance downwards migration of nitrogen, the effects may diminish before reaching groundwater depth depending on the soil and plant type^54^. Further field experimentation may shed new light on this, though this is likely to be undertaken at a much coarser resolution than in this study. The associated loss of information must therefore be weighed up.

Our research has highlighted potential weaknesses in widely used experimental approaches such as root washing that could be improved by adopting the non-invasive imaging approach used here. Prior to this study the prevailing view was that CT was limited in studies of root architecture over root washing approaches as it is not possible to segment the fine roots in CT images. Metzner *et al*.^30^ reported that only 70% of the total root length found using WinRHIZO was subsequently measured using CT analysis. Several other studies have also reported similar results, e.g. Tracy *et al*.^63^, with others also reporting they were unable to resolve roots effectively from X-ray CT images^64^. However, in this study, we found the converse with root volumes estimated from the CT images significantly greater than from WinRHIZO. We attribute this to the particular extensive fine root system of the brachiaria grasses, especially associated with palisade grass, which appear to have been excluded from the WinRhizo analysis, but which are clearly visible in the X-ray images (noting a significant time investment to acquire such data, e.g. approximately 16 hours processing time per sample). We imagine that it was most likely that the fine roots were lost in the process of root washing since the extensive fine roots can become enmeshed with the soil during the washing process despite efforts to ensure this did not happen. As X-ray CT image quality and segmentation procedures improve in the future, *in situ* imagery, such as employed here, could provide even greater accuracy and rapidity in separating roots. Moreover, the capacity to quantify 3D architecture of roots and their interactions with pore space in intact specimens provides considerably greater insight than destructive root harvesting^26^.

## Conclusions

Our research supports the hypothesis that roots influence the susceptibility of nitrate leaching from soil by directly changing soil pore structure and that this is species specific. Fine roots appear to be particularly important at reducing solute flow, primarily by producing more tortuous and complex pore networks increasing solute residency. Rooting systems significantly influence the flow of solutes through the soil, increasing nitrate leaching potential, with considerable species variability. Both maize and brachiaria increased the rate of nitrate transport through the soil whilst decreasing the total nitrate loss. Palisade grass was most effective at delaying and decreasing nitrate transport. X-ray imaging revealed considerable differences in the size of roots between the plant treatments and we hypothesise that a high volume of thin roots are more effective at reducing and delaying leaching in comparison to similar volumes of thicker roots. This is likely due to changes to the soil pore structure that they initiate. Plants with a significant network of fine roots, such as palisade grass, can generate soils with increased macroporosity, and more importantly, with increased complexity and connectivity in the pore network. This is expected to offer an advantage with respect to the retention of nitrate within soil.

Brachiaria species are increasingly being used in intercropping systems in Brazil due to the economic and environmental benefits that arise from their positive impacts on nutrient use efficiency and soil quality. Ruzigrass is currently among the most popular forage grasses due to its high biomass production and nutritional content. However, our research suggests that the use of palisade grass may be more effective from a nitrogen use efficiency perspective in modulating soil structure and retaining nitrogen. A more thorough understanding of the effects of root systems architecture on flow characteristics and solute transport in soil in the future would help support management decisions to reduce nitrate leaching and its negative environmental impacts.

## Supplementary information

Supplementary information.

## Data Availability

The datasets generated during and/or analysed during the current study are available from the corresponding author on reasonable request.
