# Supplementary material for: Brachiaria species influence nitrate transport in soil by modifying soil structure with their root system
Source: Sci Rep. 2020 Mar 19;10:5072. doi: 10.1038/s41598-020-61986-0 (PMC7081357; doi:10.1038/s41598-020-61986-0)
Supplement: Supplementary file 1 — Supplementary information. [file 41598_2020_61986_MOESM1_ESM.pdf]

Supplementary Figures

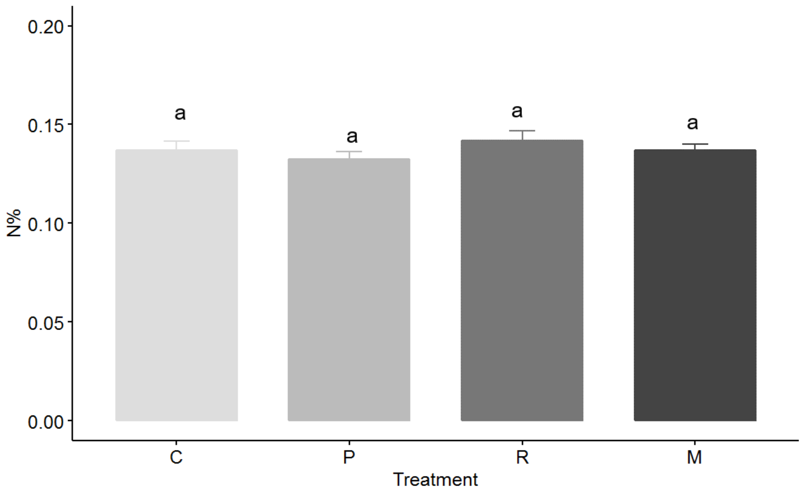

Supplementary Figure 1: Soil total nitrogen expressed as percentage (%). Error bars are one standard error from the mean. Grouping data from Tukey pairwise comparisons (95% confidence). C = Control; P = Palisade grass; R = Ruzigrass; M = Maize.

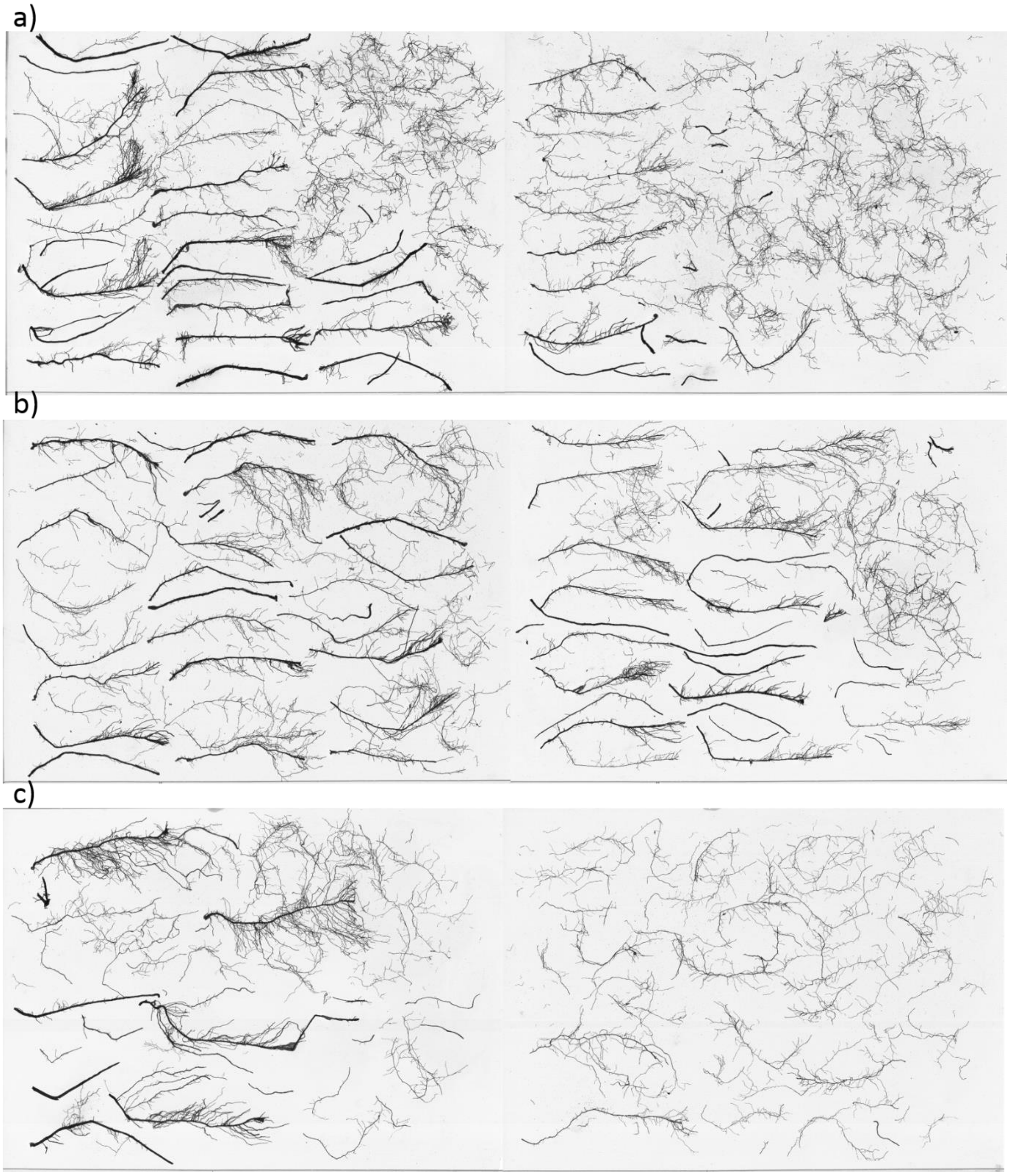

Supplementary Figure 2: Palisade grass (A), Ruzigrass (B) and Maize (C) root systems scanned by WinRHIZO.

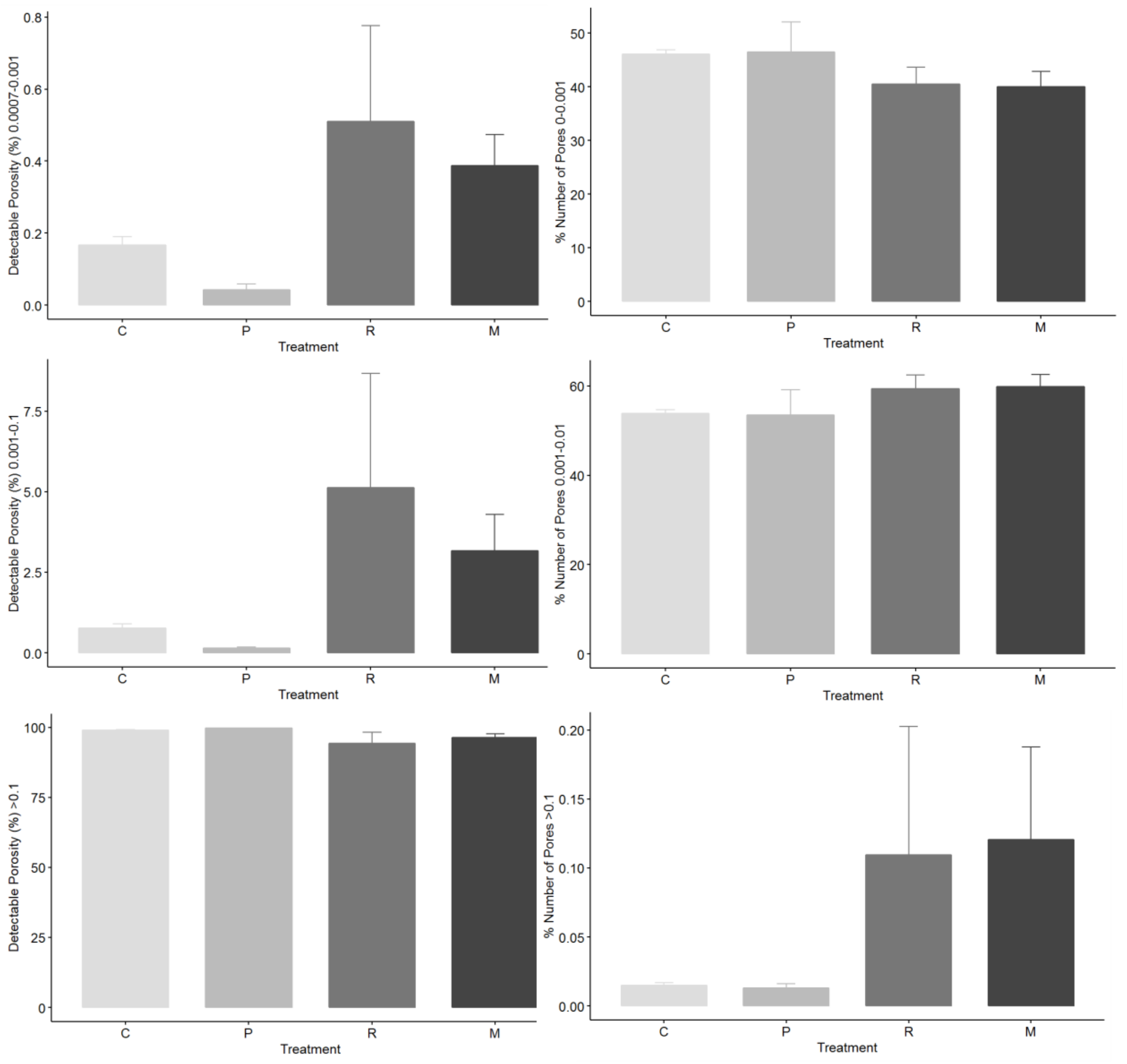

Supplementary Figure 3: Detectable porosity (mm^3) and number of pores for different pore size classes. Error bars are one standard error from the mean. Grouping data from Tukey pairwise comparisons (95% confidence). C = Control; P = Palisade grass; R = Ruzigrass; M = Maize.

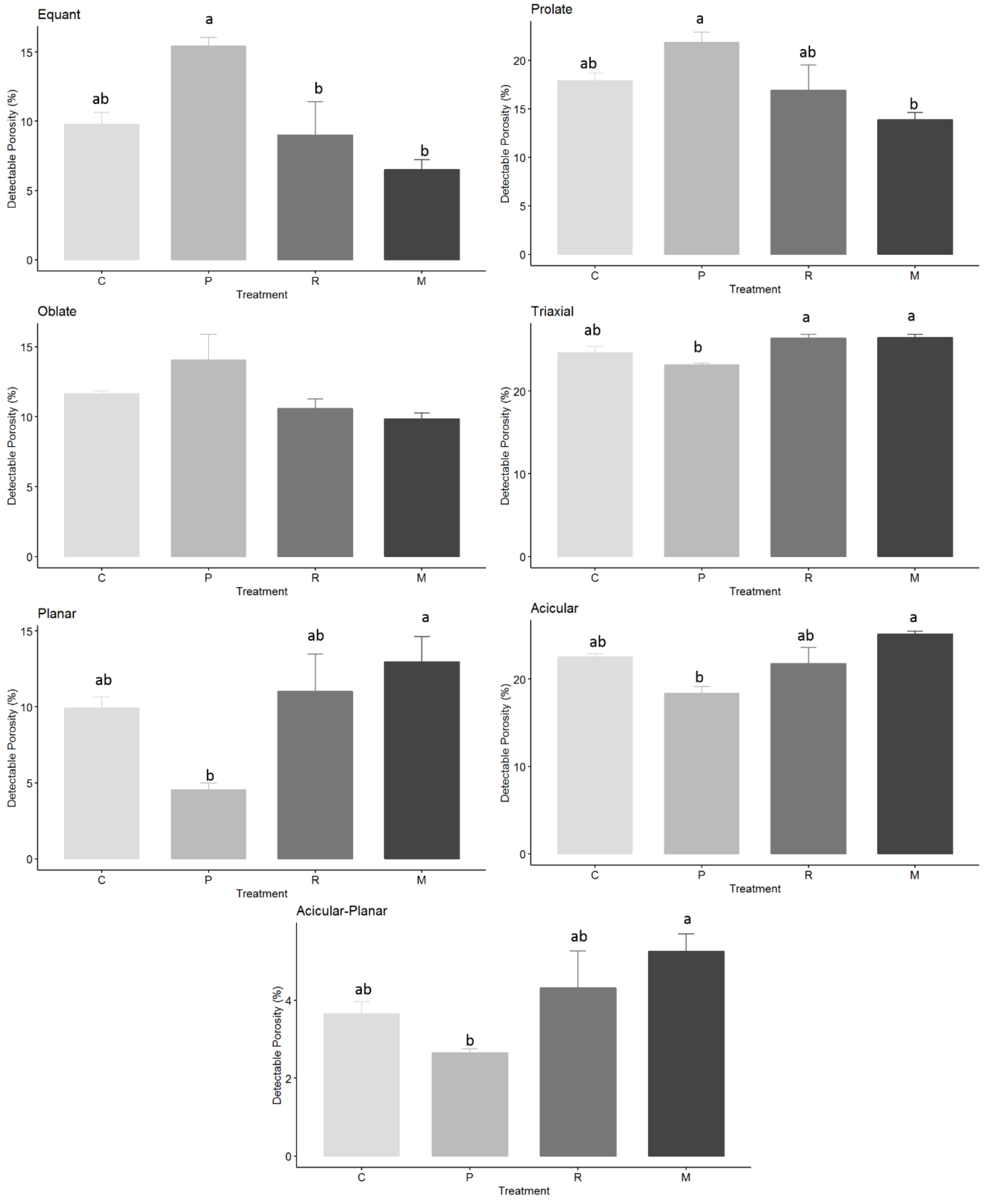

Supplementary Figure 4: Detectable porosity > 45 μm (m^3 m^{-3}) by shapes of pores. Error bars are one standard error from the mean. Grouping data from Tukey pairwise comparisons (95% confidence). C = Control; P = Palisade grass; R = Ruzigrass; M =

Maize.

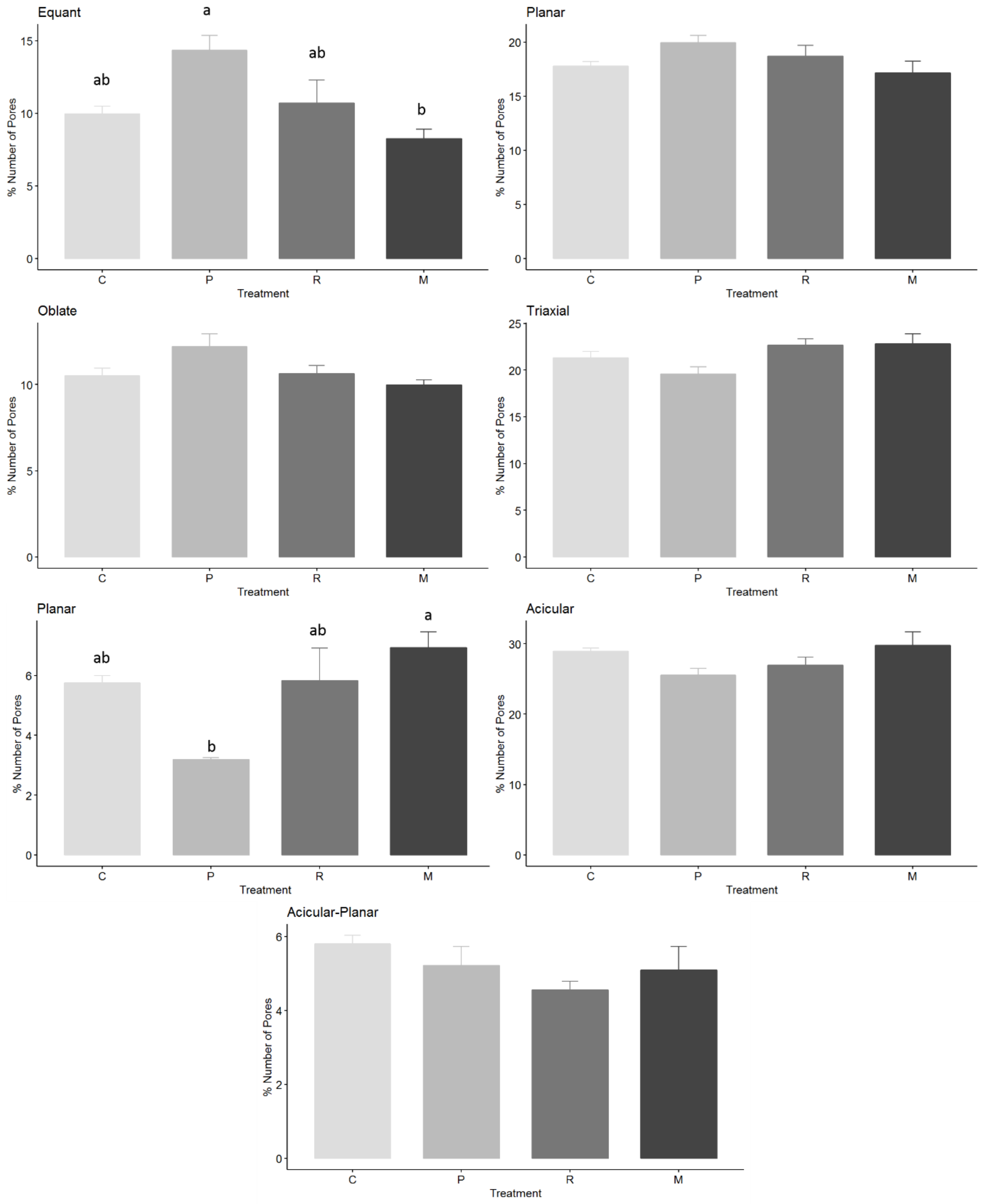

Supplementary Figure 5: Percentage of number of pores by shape. Error bars are one standard error from the mean. Grouping data from Tukey pairwise comparisons (95% confidence). C = Control; P = Palisade grass; R = Ruzigrass; M = Maize.
